# Potentially inappropriate care at the end of life in patients with glioblastoma: A single-center observational cohort study

**DOI:** 10.1093/neuonc/noag198

**Published:** 2026-09-23

**Authors:** Robin M Pieterman, Esmée K J van der Poort, Monique C M Baas-Thijssen, Matthijs van der Meulen, Erik W van Zwet, Yvette M van der Linden, Johan A F Koekkoek

**Affiliations:** Department of Neurology, Leiden University Medical Center, Leiden, The Netherlands; Department of Neurology, Leiden University Medical Center, Leiden, The Netherlands; Department of Neurology, Leiden University Medical Center, Leiden, The Netherlands; Department of Neurology, Leiden University Medical Center, Leiden, The Netherlands; Center of Medical Statistics, Leiden University Medical Center, Leiden, The Netherlands; Center of Expertise in Palliative Care, Leiden University Medical Center, Leiden, The Netherlands; Department of Radiotherapy, Leiden University Medical Center, Leiden, The Netherlands; Department of Neurology, Leiden University Medical Center, Leiden, The Netherlands

**Keywords:** advance care planning, brain tumor, end-of-life care, potentially inappropriate care, quality of life

## Abstract

**Background:**

Timely integration of palliative care is critical in the management of patients with glioblastoma. This study evaluated the frequency and determinants of potentially inappropriate care in the final 3 months of life on population level.

**Methods:**

Patient characteristics and healthcare utilization were extracted from medical records for deceased patients diagnosed with glioblastoma, who survived at least 3 months after diagnosis and received routine care at a single tertiary hospital between January 2015 and February 2026. Potentially inappropriate care was defined as the proportion of patients with any of these indicators in the final 3 months of life: ≥2 emergency department visits, ≥2 hospital admissions, >14 consecutive hospitalization days, intensive care unit admission, chemotherapy administration, or in-hospital death. Logistic regression analyses were performed to identify factors associated with receiving potentially inappropriate care.

**Results:**

The cohort included 310 patients (median age 66 [IQR 56-72], 60% male), of whom 22% (*n* = 67/310) received potentially inappropriate care in the final 3 months of life. Chemotherapy was the main driver (*n* = 58/67, 87%), followed by ≥2 hospital admissions (*n* = 10/67, 15%) and in-hospital death (*n* = 9/67, 13%). Older patients (OR 0.97, 95% CI, 0.94-0.99) or those with an unmethylated O^6^-methylguanine-DNA methyltransferase (MGMT) promoter (OR 0.42, 95% CI, 0.23-0.75) were less likely to receive potentially inappropriate care.

**Conclusion:**

One in 5 patients with glioblastoma received potentially inappropriate care at the end of life, predominantly through chemotherapy. Younger patients and those with methylated MGMT promoters were most affected. Identification of patients at risk may support targeted palliative care interventions.

Key Points

- One in 5 patients received potentially inappropriate care at the end of life.
- Chemotherapy was the main driver of potentially inappropriate care.
- Identifying patients close to death is key to avoid inappropriate treatment.

Importance of the Study

In patients with glioblastoma, health-related quality of life eventually becomes the main focus of care, as the benefits of disease-targeted treatments may no longer outweigh their negative effects toward the end-of-life phase. However, the prevalence and determinants of potentially inappropriate care at the end of life, including chemotherapy, hospital admissions, and in-hospital death, are currently unclear and, if available, lack clinical details. This study evaluated the medical records of deceased patients with glioblastoma and found that, on population-level, 1 in 5 received potentially inappropriate care in the final 3 months and 1 in 17 in the final 30 days of life; this is considerably lower compared to other cancer types. Patients who received potentially inappropriate care were younger and had a methylated O^6^-methylguanine-DNA methyltransferase (MGMT) promoter, and the main determinant was the administration of chemotherapy. These insights may facilitate early identification of patients at risk and support earlier, more targeted palliative care.

Glioblastoma, isocitrate dehydrogenase (IDH) wildtype, WHO grade 4, is the most common malignant primary brain tumor in adults and carries a poor prognosis. Despite multimodal treatment with surgery, chemotherapy, and/or radiotherapy, the median survival is 12-17 months.^1^ Progressive cognitive deficits impair communication and medical decision making in patients with glioblastoma,^2^ with less than 50% of patients being able to make decisions in the final weeks of life.^2-6^ The combination of incurable cancer, a poor prognosis, and neurological decline also places a substantial burden on informal caregivers, often relatives of patients, who report higher distress^2^^,^^3^^,^^7-9^ and support needs compared to caregivers of other patients receiving palliative care.^9^ These features make timely advance care planning critical in the management of patients with glioblastoma, as recommended in international guidelines.^10^^,^^11^

As patients with glioblastoma near the end of their lives, the goals of care gradually shift from disease control toward primarily maintaining health-related quality of life (HRQoL) as the benefits of disease-targeted treatment may no longer outweigh their negative effects within a limited life expectancy.^12-15^ Care at the end of life ideally aligns with the preferences of patients and their relatives regarding the nature of care, ie goal-concordant care. Goal-concordant care can be established through advance care planning, a major component of palliative care aimed to align future care with the preferences, wishes, and values of patients and their relatives, while patients are still able to participate in conversations.^16^^,^^17^ In patients with glioblastoma, advance care planning and early palliative care improve HRQoL, reduce relocations during the final three months of life, and increase the quality of care at the end of life.^18^^,^^19^

Establishing whether the care patients received near the end of life was goal-concordant can be challenging, as no validated methods exist.^20^^,^^21^ Population-based indicators for potentially inappropriate care can be used to determine if patients received care that would be considered appropriate or inappropriate. Recommended indicators for potentially inappropriate care include a high number of emergency department (ED) visits, hospital admissions, days spent in the intensive care unit (ICU), administration of chemotherapy, and death in an acute care setting.^13-15^^,^^22^ Reducing the rate of potentially inappropriate care has been associated with better outcomes for both patients and relatives.^23^^,^^24^ In other advanced cancer populations, early integration of palliative care has been associated with less aggressive care at the end of life, including fewer hospital admissions and less chemotherapy in the final weeks of life.^25^ Whether palliative care involvement similarly reduces the receipt of potentially inappropriate care in patients with glioblastoma has not been established.

It remains unclear how many patients with glioblastoma receive potentially inappropriate care at the end of life, and existing population-based data are limited and often lack detailed clinical information.^26^ However, insight into the proportion of patients receiving potentially inappropriate care and the patient characteristics associated with it may help identify those at risk and may support earlier, more targeted palliative care interventions. This study aimed to determine the proportion of patients with glioblastoma receiving potentially inappropriate care in the final 3 months of life on a population-level, and to explore the influence of patient characteristics and HRQoL on its receipt.

## Methods

### Study Design and Ethics

This single-center retrospective observational cohort study was conducted at Leiden University Medical Center (LUMC) in The Netherlands. The study was approved by the medical ethics research committee of LUMC (reference number 25-3048). The requirement for informed consent was waived in accordance with the Dutch Medical Research Involving Human Subjects Act.

### Study Population and Data Sources

Patient electronic medical records were searched with Ctcue (IQVIA, version 4.17.1) to identify eligible patients and extract relevant data on patient characteristics, health care utilization, and HRQoL. CTcue is a privacy-by-design tool that enables healthcare organizations to use machine learning to review pseudonymized structured and unstructured data in electronic medical records. Patients were eligible if they (1) were diagnosed with glioblastoma according to the World Health Organization 2021 classification of tumors in the central nervous system^27^ or were highly suspected of having glioblastoma, (2) received their main glioblastoma care at the LUMC outpatient clinic between January 2015 and February 2026, (3) survived for at least 3 months after diagnosis, (4) were at least 18 years old at the time of diagnosis, and (5) had no active objection to the use of their data for scientific research. Main glioblastoma care was defined as (1) at least one consultation at the outpatient neuro-oncology clinic between January 2015 and February 2026 and (2) a histopathological diagnosis of glioblastoma. In the absence of a local histopathological diagnosis, patients remained eligible if the pathological diagnosis was obtained in another hospital and documented in the medical record; if the pathological diagnosis was not feasible or desired given the patient’s clinical status and/or preferences but radiological imaging was highly suggestive of glioblastoma; or if technical issues had impaired pathological diagnosis but radiological imaging was highly suggestive of glioblastoma. Patients were excluded from the cohort if any of the relevant patient characteristics were unavailable, except for missing O^6^-methylguanine-DNA methyltransferase (MGMT) promoter methylation status or unavailable HRQoL data.

### Variables

#### Cohort Characteristics

For eligible patients, age at diagnosis, sex (male/female), date of death, Karnofsky performance score (KPS; first measurement), and pathological diagnosis were extracted. For the pathological diagnosis, we obtained tumor localization, MGMT promoter methylation status, and the type of surgical treatment for glioblastoma (biopsy-only, resection, or no surgery). All these data were extracted from 2 months before diagnosis (as derived from the date of the pathology report, or, if no pathology report was available, from the date of the first consultation) until death. Survival time from diagnosis (in months) was calculated as the interval between the date of pathological diagnosis and the date of death.

#### Potentially Inappropriate Care at the End of Life

The primary outcome was the percentage of patients receiving potentially inappropriate care in the last 3 months of life. In line with previous international studies, potentially inappropriate care was defined as the percentage of patients with at least one of the following indicators: ≥2 ED visits, ≥2 hospital admissions, >14 consecutive days of hospital and/or ICU admission, ≥1 ICU day, ≥1 chemotherapy administration, or in-hospital death.^14^^,^^28-30^ Potentially inappropriate care was determined for both the last 3 months of life and the last 30 days of life. These time frames were chosen as, in retrospect, the final 3 months can generally be considered the end-of-life phase in patients with glioblastoma.^2^^,^^10^^,^^31^ Including the last 30 days of life allows the identification of changes in provided care over time and comparison with other studies on potentially inappropriate care at the end of life in patients with cancer and with national normative data.

#### Health Care Utilization

Health care utilization was assessed from the care activities registered in the electronic medical records over the same period defined above. Care activities are uniformly and systematically documented for health care administration purposes and are not based on self-reported data. The following health care activities were of specific interest for this study: chemotherapy (including the specific drug), radiotherapy, ED visits, hospital admissions, ICU admissions, and in-hospital death. All care activities were included, regardless of their reason or duration.

#### HRQoL

Health-related quality of life was assessed through the EORTC QLQ-C30 (version 3.0, in Dutch) and the EORTC QLQ-BN20 (in Dutch). The EORTC QLQ-C30 is a core questionnaire for patients with cancer consisting of 30 items that comprise 5 functional scales, 3 symptom scales, 6 single items, and a global health status/QoL scale.^32^ The EORTC QLQ-BN20 is a supplement to the QLQ-C30, containing 20 items that focus on brain tumor-specific symptoms and comprises 4 symptom scales and 7 single items.^33^ For both questionnaires, items are scored on a 4-point Likert scale ranging from “not at all” to “very much,” except for the global health status/QoL scale, which is scored on a 7-point Likert scale. Raw scores were converted into a 0-100 scale following the EORTC scoring manual.^34^ For the EORTC QLQ-C30, a summary score was calculated.^35^ Higher scores on functional scales indicate better functioning, while higher scores on symptom scores indicate more symptoms.

### Statistical Principles

All statistical analyses were performed in R studio (version 2025.05.0, build 496).^36^

#### Descriptive Statistics

Binary variables were described using number (*n*) and percentages (%). The distribution of continuous variables was determined using Q-Q plots and the Shapiro-Wilk test (*P *< .05 indicated non-normal distribution). Normally distributed continuous variables were described using mean and standard deviation (SD), while non-normally distributed continuous variables were described using the median and interquartile range (IQR). For HRQoL, mean and SD were reported regardless of distribution.

#### Survival Analysis

The survival in months from diagnosis until death was plotted in a Kaplan-Meier curve, and the median and 95% confidence intervals (CI) are provided.

#### Factors Associated With the Proportion of Patients With Potentially Inappropriate Care

To analyze the association between patient characteristics and the receipt of potentially inappropriate care in the last 3 months of life, we performed a logistic regression. All patients in the cohort were included in the model, except those without determined MGMT promoter methylation status. The main logistic regression model included sex (binary), age at diagnosis (continuous), MGMT promoter methylation status (binary), and KPS (continuous) (forced entry method). In addition, tumor localization (7 regions) and type of surgery (as biopsy, resection, or none) were added to the model only if their addition improved the model, as assessed through the Akaike information criterion (AIC). A separate logistic regression was performed on the receipt of chemotherapy at the end-of-life, as the largest contributor to potentially inappropriate care. This second model was set up using the same approach as the main model. Assumptions for logistic regression, including linearity of the logit, independence of errors, multicollinearity, influential outliers, and sufficient sample size, were assessed using appropriate diagnostic procedures. Regression outcomes are presented as odds ratios (OR) and 95% CI. ORs indicated significant effects if the CI does not overlap with an OR of 1.

#### Sensitivity Analysis on the Definition of Potentially Inappropriate Care

A sensitivity analysis assessed the stability of the proportion of patients receiving potentially inappropriate care in the last 3 months of life by varying the definition for 3 indicators: (1) removing chemotherapy administration as indicator, (2) changing ≥2 ED visits to ≥1 ED visit, and (3) changing ≥2 hospital admissions to ≥1 hospital admission.

#### HRQoL and the Receipt of Potentially Inappropriate Care

For a subset of HRQoL scales, the first available assessment from diagnosis was compared between patients who received potentially inappropriate care and those who did not. Relevant HRQoL scales were selected based on the most important scales defined by RANO-PRO^37^ and on clinical relevance as determined by the study authors. Included were the global health (quality of life), all functioning scales (physical, role, emotional, cognitive, and social), and the summary score^35^ from the EORTC QLQ-C30, and the communication deficits and seizure scales from the EORTC QLQ-BN20. The distribution of relevant mean scores was compared between patients who received potentially inappropriate care in the final 3 months of life with those who received potentially appropriate care in the final 3 months of life. Potential clinically relevant differences^38^ are indicated, although no formal statistical testing was performed.

## Results

### Cohort Description

The cohort comprised 310 eligible patients with glioblastoma. The median age was 66 years (IQR 56-72), 60% (*n* = 185/310) were male, and the median survival from diagnosis was 11 months (IQR 7-18). Patient characteristics are described in Table 1, and survival from diagnosis is shown in Appendix Figure S1. Generalist palliative care, provided by the neuro-oncologist and neuro-oncology nurse, was provided to all patients. Additionally, 25 patients received a consultation with the specialist palliative care team, which took place in the final 30 days of life for 36% (*n* = 9/25) of patients, in the final 1-3 months for 32% (*n* = 8/25), or earlier in 32% (*n* = 8/25) of patients.

**Table 1.** Patient characteristics

	Overall	Final 3 months		Final 30 days	
		Potentially appropriate care	Potentially inappropriate care	Potentially appropriate care	Potentially inappropriate care
	*n *= 310 (100%)	*n* = 243 (78%)	*n* = 67 (22%)	*n* = 291 (94%)	*n* = 19 (6%)
**Male gender [*n* (%)]**	185 (60%)	142 (58%)	43 (64%)	175 (60%)	10 (53%)
**Age at diagnosis, in years [median (IQR)]**	66 (56-72)	68 (58-73)	60 (55-71)	67 (57-72)	58 (52-68)
**Baseline KPS [*n* (%)]**					
KPS > =70	270 (87%)	209 (86%)	61 (91%)	254 (87%)	16 (84%)
KPS <70	40 (13%)	34 (14%)	6 (9.0%)	37 (13%)	3 (16%)
**MGMT promoter methylation [*n* (%)]**					
Methylated	106 (34%)	74 (30%)	32 (48%)	98 (34%)	8 (42%)
Non-methylated	196 (63%)	162 (67%)	34 (51%)	186 (64%)	10 (53%)
Not determined	8 (2.6%)	7 (2.9%)	1 (1.5%)	7 (2.4%)	1 (5.3%)
**Localization [*n* (%)]**					
Brain stem	5 (1.6%)	2 (0.8%)	3 (4.5%)	4 (1.4%)	1 (5.3%)
Frontal	103 (33%)	78 (32%)	25 (37%)	95 (33%)	8 (42%)
Midline	8 (2.6%)	6 (2.5%)	2 (3.0%)	7 (2.4%)	1 (5.3%)
Multifocal	1 (0.3%)	1 (0.4%)	0 (0%)	1 (0.3%)	0 (0%)
Occipital	13 (4.2%)	11 (4.5%)	2 (3.0%)	13 (4.5%)	0 (0%)
Parietal	70 (23%)	56 (23%)	14 (21%)	65 (22%)	5 (26%)
Temporal	110 (35%)	89 (37%)	21 (31%)	106 (36%)	4 (21%)
**Surgery [*n* (%)]**					
Biopsy only	70 (23%)	54 (22%)	16 (24%)	64 (22%)	6 (32%)
No surgery	2 (0.6%)	2 (0.8%)	0 (0%)	2 (0.7%)	0 (0%)
Resection	238 (77%)	187 (77%)	51 (76%)	225 (77%)	13 (68%)
**Treatment [*n* (%)]**					
Best supportive care	27 (8.7%)	27 (11%)	0 (0%)	27 (9.3%)	0 (0%)
Chemotherapy	13 (4.2%)	6 (2.5%)	7 (10%)	12 (4.1%)	1 (5.3%)
Chemo- and radiotherapy	194 (63%)	141 (58%)	53 (79%)	181 (62%)	13 (68%)
Radiotherapy	76 (25%)	69 (28%)	7 (10%)	71 (24%)	5 (26%)
**Type of chemotherapy (*n* = 207) [*n* (%)]**					
Lomustine and temozolomide	33 (16%)	26 (18%)	7 (12%)	33 (17%)	0 (0%)
Temozolomide	174 (84%)	121 (82%)	53 (88%)	160 (83%)	14 (100%)

Table showing the patient characteristics of those included in the cohort. Displayed are *n* (%) and median (IQR), where appropriate.

### Potentially Inappropriate Care at the End of Life

In the final 3 months of life, 22% (*n* = 67/310) of patients received potentially inappropriate care. This fell to 6% (*n* = 19/310) in the final 30 days of life. Most patients met only 1 of the 6 indicators of potentially inappropriate care (*n* = 55/67 [82%] in the final 3 months and *n* = 14/19 [74%] in the final 30 days of life). However, some patients met 3 or more indicators (*n* = 6/67 [9%] in the final 3 months and *n* = 3/19 [16%] in the final 30 days of life) (Figure 1A). Among the patients meeting more than 1 indicator, the combinations were diverse (Appendix Table S1). In the final 3 months of life, chemotherapy was the largest driver for potentially inappropriate care (*n* = 58/67 [87%]), followed by ≥2 hospital admissions (*n* = 10/67 [15%]), in-hospital death (*n* = 9/67 [13%]), hospital admission for >14 consecutive days and ≥2 ED visits (both *n* = 5/67 [7%]), and ICU admission (*n* = 3/67 [4%]). In the final 30 days of life, chemotherapy and in-hospital death (both *n* = 9/19 [47%]) were the main drivers for potentially inappropriate care, followed by hospital admission for >14 consecutive days (*n* = 4/19 [21%]), ICU admission (*n* = 3/19 [16%]), and ≥2 hospital admissions and ≥2 ED visits (both *n* = 1/19 [6%]) (Figure 1B). In patients who had specialized palliative care consult, 24% (*n *= 6/25) received potentially inappropriate care in the final 3 months and 12% (*n* = 3/25) in the final 30 days of life.

**Figure 1.**
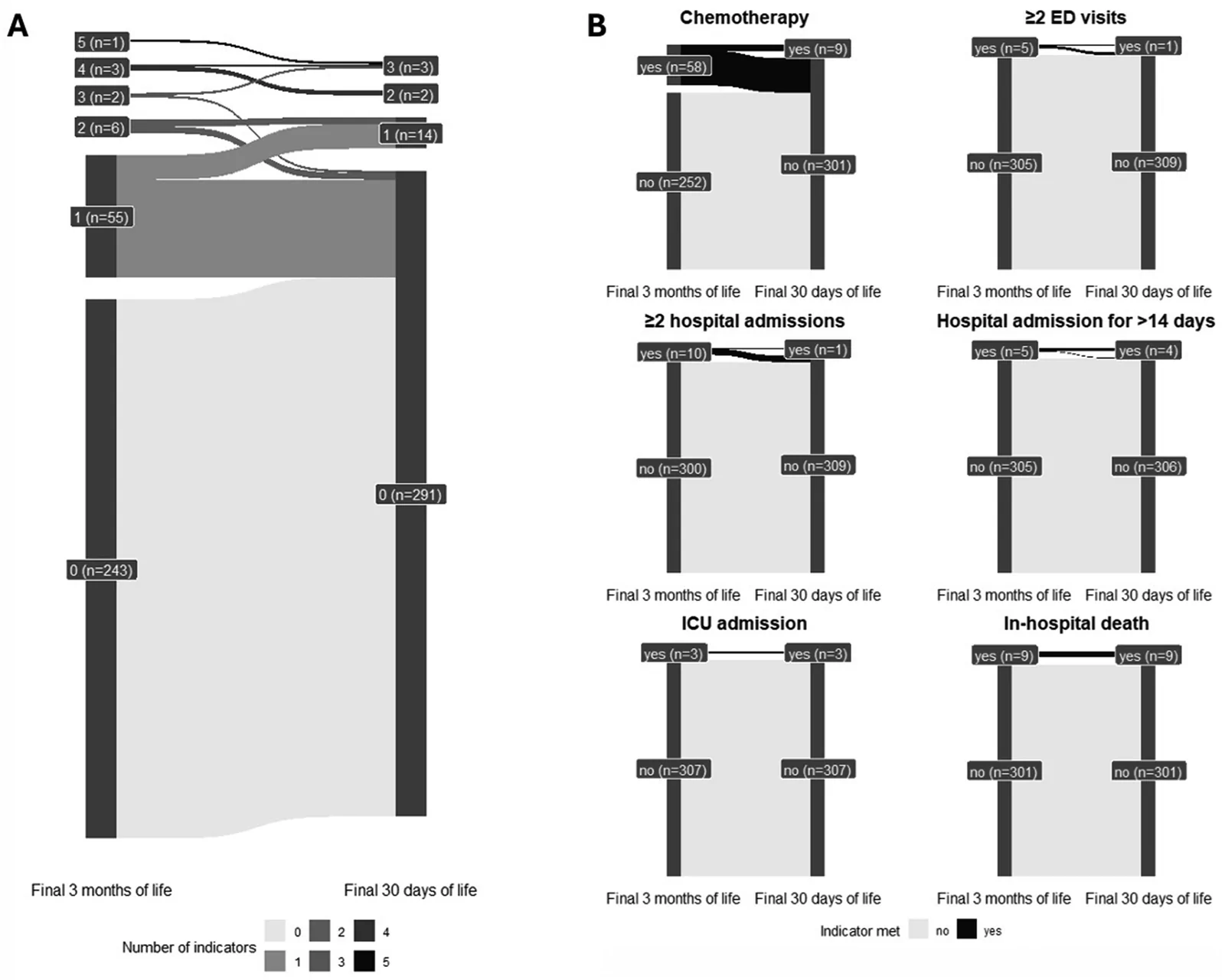
Indicators of potentially inappropriate care at the end of life. Sankey diagrams showing the (A) number of indicators per patient met in the final 3 months and the final 30 days of life, and (B) the number of patients per specific indicator in the final 3 months and final 30 days of life. Patients with 0 indicators, shown in light grey, did not meet any indicator of potentially inappropriate care at the end of life.

In the sensitivity analysis, excluding chemotherapy from the list of indicators led to a decrease of 16 percentage points in patients receiving potentially inappropriate care in the last 3 months. Likewise, a reduction in the required number of ED visits and hospital admissions to at least 1 was associated with a 10 percentage point increase in patients receiving potentially inappropriate care in the last 3 months (Table 2).

**Table 2.** Sensitivity analysis on the definition of potentially inappropriate care at the end of life

	Final 3 months		Final 30 days	
	Potentially appropriate care [*n* (%)]	Potentially inappropriate care [*n* (%)]	Potentially appropriate care [*n* (%)]	Potentially inappropriate care [*n* (%)]
Original definition^a^	243 (78%)	67 (22%)	291 (94%)	19 (6%)
Definition^a^ without chemotherapy (instead of with chemotherapy)	292 (94%)	18 (6%)	299 (96%)	11 (4%)
Definition^a^ with ≥1 ED visit (instead of ≥2)	210 (68%)	100 (32%)	276 (89%)	34 (11%)
Definition^a^ with ≥1 hospital admission (instead of ≥2)	212 (68%)	98 (32%)	274 (88%)	36 (12%)

Displayed for the whole cohort (*n* = 310). Displayed are *n* (%).

a Patients received potentially inappropriate care at the end of life if at least one of the following indicators was met: ≥2 ED visits, ≥2 hospital admissions, >14 consecutive days of hospital and/or ICU admission, ≥1 ICU day, ≥1 chemotherapy administration, or in-hospital death.

### Characteristics Associated With Potentially Inappropriate Care in the Final 3 Months

Multivariable logistic regression analysis was performed to identify patient characteristics associated with the receipt of any potentially inappropriate care (Model A) and with the administration of chemotherapy (Model B) in the final 3 months of life. Both models included sex, age at diagnosis, MGMT promoter methylation status, and KPS at baseline. None of the additional parameters tested improved model fit, as described in the Methods section, and hence were not included in the final models. The models were run on *n* = 302 patients in the cohort, as *n* = 8 patients were excluded due to the lack of MGMT methylation status. There were no other missing data. Patients who were older at diagnosis (OR 0.97, 95% CI, 0.94-0.99) and those with an unmethylated MGMT promoter (OR 0.42, 95% CI, 0.23-0.75) were less likely to receive potentially inappropriate care (Model A; Table 3). Patients who were older (OR 0.96, 95% CI, 0.94-0.99) and those with an unmethylated MGMT promoter (OR 0.30, 95% CI, 0.16-0.56) were less likely to receive chemotherapy (Model B; Table 3). Sex and KPS were not associated with the receipt of potentially inappropriate care nor chemotherapy in the final 3 months of life.

**Table 3.** Logistic regression for potentially inappropriate care in the final 3 months of life

	OR	95% CI
**Model A (potentially inappropriate care)**		
Male gender	1.23	0.68-2.23
Age at diagnosis	0.97	0.94-0.99
MGMT promoter non-methylated	0.42	0.23-0.75
Baseline KPS	0.99	0.97-1.01
**Model B (chemotherapy only)**		
Male gender	1.35	0.71-2.55
Age at diagnosis	0.96	0.94-0.99
MGMT promoter non-methylated	0.30	0.16-0.56
Baseline KPS	1.00	0.97-1.03

ORs indicated significant effects if the CI does not overlap with an OR of 1.

### HRQoL and Potentially Inappropriate Care in the Final 3 Months

Patient-reported outcomes were available for the period from January 2022 through February 2026, during which 66 patients completed at least 1 of the HRQoL questionnaires. Of these patients, 39% (*n* = 26/66) answered the questionnaire within 2 months of diagnosis, 27% (*n* = 18/66) between 2 and 6 months of diagnosis, and 33% (*n* = 22/66) at least 6 months after diagnosis. Baseline characteristics of these patients are displayed in Appendix Table S2. Compared to the full cohort, patients with HRQoL data available had slightly higher baseline KPS (92% versus 87% with KPS > = 70), and all of these patients received radio- and/or chemotherapy (0% versus 8.7% of patients received best supportive care). Among the patients with HRQoL data available, 18% (*n* = 12/66) received potentially inappropriate care in the final 3 months of life and 3% (*n* = 2/66) in the final 30 days of life. Of these patients, 65 completed the EORTC QLQ-C30, and 62 completed the EORTC QLQ-BN20. The HRQoL scores are displayed in Table 4 and Figure 2. Patients who received potentially inappropriate care had higher global health scores (mean change of 9) and better role, emotional, and social functioning scores (mean change of 21, 7, and 10, respectively) (not statistically tested).

**Figure 2.**
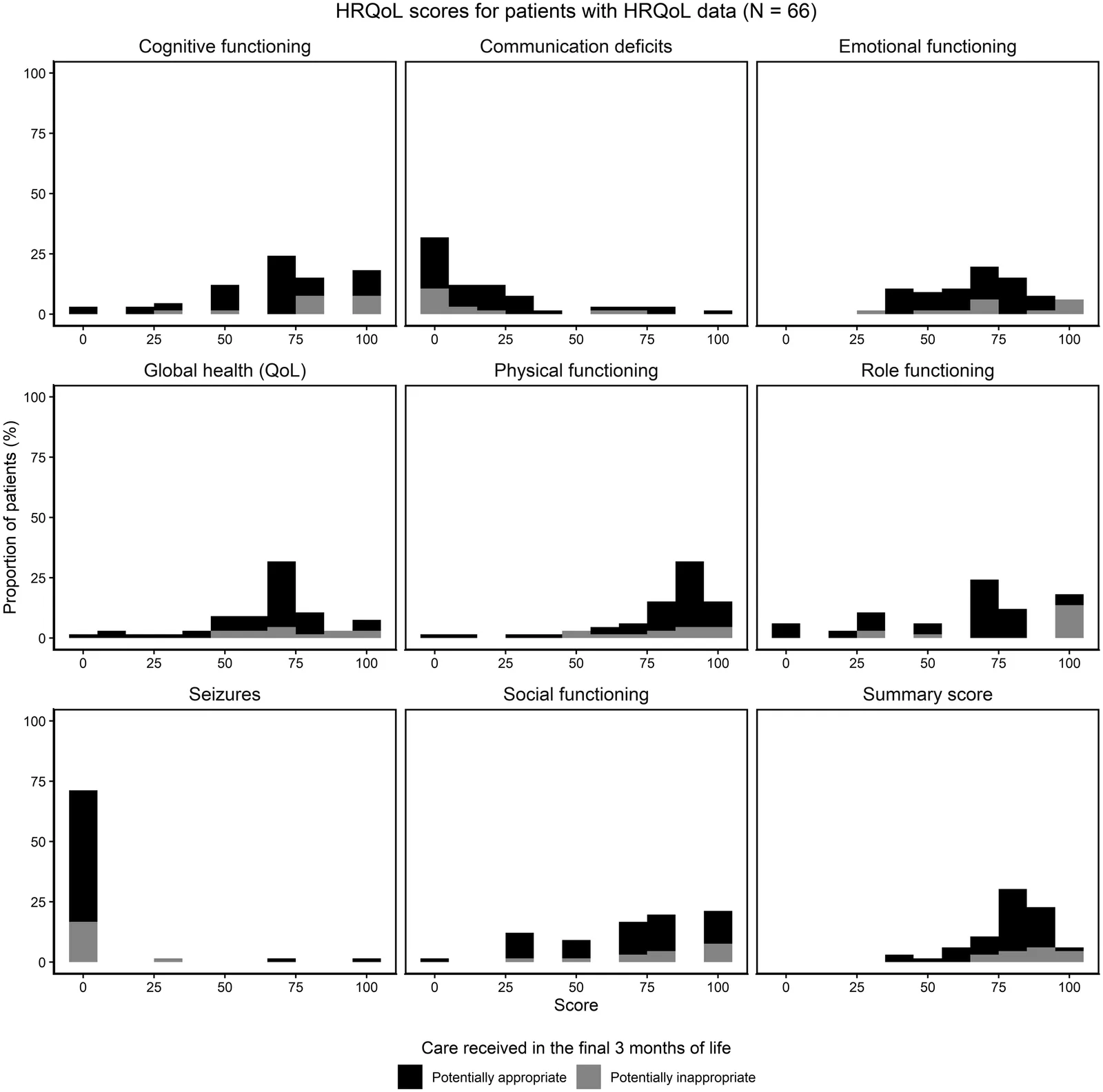
Scores for selected HRQoL items. Histograms showing the distribution of scores from selected HRQoL items for patients with at least one answered HRQoL survey (*n* = 66). In dark grey the patients who received potentially appropriate care, with a light grey overlay showing the scores in patients who received potentially inappropriate care. The communication deficits and seizure items were obtained through the EORTC QLQ-BN20, and all others through the EORTC QLQ-C30. Higher scores on functional scales indicate better functioning, while higher scores on symptom scores indicate more symptoms.

**Table 4.** Descriptive statistics for selected HRQoL scores

	Patients with HRQoL data	Final 3 months	
		Potentially appropriate care	Potentially inappropriate care
**HRQoL data available [*n* (%)]**	**66 (100%)**	**54 (82%)**	**12 (18%)**
**EORTC QLQ-C30 (*n *= 65) [mean (SD)]**			
Summary score	81 (14)	79 (14)	87 (11)
Global health (QoL)^a^	67 (22)	65 (22)	74 (19)
Physical functioning	81 (21)	81 (22)	81 (17)
Role functioning^a^	68 (31)	64 (30)	85 (28)
Emotional functioning^a^	70 (19)	69 (19)	76 (23)
Cognitive functioning^a^	71 (26)	69 (26)	83 (21)
Social functioning^a^	73 (25)	71 (25)	81 (22)
**EORTC QLQ-BN20 (*n* = 62) [mean (SD)]**			
Communication deficits	18 (25)	20 (25)	14 (23)
Seizures	4 (16)	4 (17)	3 (10)

HRQoL scores for patients with available data are displayed using mean (SD).

a Indicates scales domains with a potentially clinically relevant difference (not formally tested). Higher scores on functional scales indicate better functioning, while higher scores on symptom scores indicate more symptoms.

## Discussion

This retrospective cohort study used medical records to examine the end-of-life care of 310 patients with glioblastoma treated at a single institution. Six population-based indicators were used to determine whether the care at the end of life was potentially appropriate or inappropriate: ≥2 ED visits, ≥2 hospital admissions, >14 consecutive days of hospital and/or ICU admission, ≥1 ICU day, ≥1 chemotherapy administration, and/or in-hospital death. Based on these population-based indicators, 1 in 5 patients received potentially inappropriate care in the final 3 months of life; these patients were generally younger and more often had glioblastoma with a methylated MGMT promoter. While a small number of patients met 3 or more indicators, the main driver of potentially inappropriate care turned out to be the administration of chemotherapy. A sensitivity analysis revealed that removing chemotherapy as an indicator reduced the proportion of patients with potentially inappropriate care from 22% to 6% in the final 3 months of life, whereas 1 in 3 patients were admitted to the hospital or visited the ED at least once in the final 3 months of life. In the final 30 days of life, 1 in 17 patients received potentially inappropriate care, although some patients still met more than 1 of the 6 indicators.

The proportion of patients who received potentially inappropriate care in our cohort was smaller than that reported in studies in patients with other cancers using population-based approaches. In the Netherlands, studies using the same definition of potentially inappropriate care found that 24%-34% of patients with cancer received potentially inappropriate care in the final 30 days of life, with in-hospital death as the main driver (14%-20% of patients).^28^^,^^39^ However, these studies included all care patients received across different care providers and were not limited to a single hospital like in our study. Studies in patients with glioblastoma across Western countries, using varying definitions of potentially inappropriate care, found that 31%-59% of patients received potentially inappropriate care in the final 30 days of life.^26^^,^^40^^,^^41^ One study in Finland, using a similar setting as in our study, found that 21% of patients received chemotherapy, 22% visited the ED, and 17% were hospitalized in the final 30 days of life, and 8% of patients died in the hospital.^40^ The median overall survival in our cohort was 11 months, which is likely explained by the inclusion of deceased patients only, thereby excluding longer-term survivors diagnosed in later years.

In our cohort, younger patients and those with a methylated MGMT promoter were more likely to receive potentially inappropriate care at the end of life. Younger patients may be fitter and may have a stronger preference for treatment or participation in clinical trials. The association between younger age and receipt of potentially inappropriate care may also reflect a tendency toward more intensive treatment in younger patients, as clinicians and patients may be more inclined to pursue life-prolonging treatment despite limited expected benefit. Consequently, care that is recognized as potentially inappropriate in older patients may be perceived as more acceptable in younger patients, although certain care may still be disproportionate in the context of limited prognosis. The association with MGMT promoter methylation may be related to the expected benefit of temozolomide, the main chemotherapeutic agent used in the management of glioblastoma. Patients with a methylated MGMT promoter tend to derive greater benefit from temozolomide, compared to patients with an unmethylated MGMT promoter.^42-46^ Temozolomide can be taken orally and is generally well tolerated,^47^ and hence the threshold to continue its use into the final months of life may be lower. These findings raise the question whether chemotherapy should always be considered inappropriate at the end of life in patients with glioblastoma, particularly as it was the largest driver of potentially inappropriate care in our cohort and its removal as an indicator lowered the proportion substantially. Nonetheless, while administration of chemotherapy in the final 3 months of life may be appropriate in patients with glioblastoma, its continued use should be reassessed as death approaches.

There is currently no universal method to define appropriate or goal-concordant care at the end of life.^20^^,^^21^ In our study, internationally recommended and reported population-based indicators were used to determine the appropriateness of care at the end of life.^14^^,^^22^^,^^28-30^^,^^41^^,^^48-50^ This population-based approach of determining appropriateness of care assumes that aggregated data accurately reflect individual patients’ goals and priorities, which might not be true.^51^ The approach also does not account for the invasiveness or burden of treatments, as continuation of the well-tolerated chemotherapy drug temozolomide within the final 30 days of life poses a different scenario compared to repeated ICU and ED visits in this time period. Future population-based indicators may want to include an invasiveness measure along with the dichotomous indicator. Another drawback of the population-based indicators for potentially inappropriate care at the end of life is the definition of the end-of-life phase. The end-of-life phase can only be fully delineated retrospectively in an individual patient, while population-based indicators require a common time frame such as the 3 months used in our study. The specific time frame of 3 months was chosen as it can generally, in retrospect, be considered the end-of-life phase in patients with glioblastoma.^2^^,^^10^^,^^31^ However, the time frame cannot be applied prospectively at the bedside, where the proximity of death is uncertain, and it therefore does not by itself indicate when a given treatment decision should be reconsidered. Related to this 3-month time frame is the inclusion criterion in our cohort that patients needed to survive at least 3 months from diagnosis. This does indeed exclude patients with shorter survival, who may be especially prone to visit the ED or be hospitalized to manage acute symptoms and may not have received palliative care due to the rapid disease course. Nonetheless, the population-based indicator for potentially inappropriate care does allow pragmatic research in a large number of patients, follow-up over time, and a comparison with other cohorts. Alternative methods to investigate the appropriateness of care are no perfect alternatives either, as patient- and caregiver-reported assessments are affected by selection and recall bias, while longitudinal methods comparing documented goals with received care are underdeveloped, as the documentation of care goals is usually inconsistent and fragmented across care facilities.^51^ In our cohort, there was no systematic recording of advance care planning documentation available, so direct comparison of recorded individual preferences with actual received care was not possible.

This study is part of a larger project of improving palliative care in patients with glioblastoma across all disease stages. There are some limitations to the current study, which are lessons for future studies. First, there is a lack of insight into health care utilization of patients at other institutions. While all care that patients receive within LUMC was captured from medical records, patients might have sought emergency care elsewhere. Hence, there may be an underestimation of the true proportion of patients that have received potentially inappropriate care. Second, bevacizumab was not captured as systemic anticancer treatment in this study. In the Netherlands, bevacizumab is not registered for recurrent glioblastoma. Its use is restricted to dexamethasone-refractory cerebral radiation necrosis, so it was registered in only a few patients. In countries where bevacizumab is an approved treatment option for recurrent glioblastoma, the proportion of patients receiving systemic treatment near the end of life may be higher than observed in our cohort. As with temozolomide, the use of bevacizumab may nonetheless be appropriate late in the disease course, as long as it contributes to symptom control and maintenance of quality of life.^52^ Third, HRQoL data was sparse and collected at variable time points across the disease trajectory. While all patients were encouraged to answer several HRQoL questionnaires at set time intervals, generally during the first outpatient clinic visit and every 3 months thereafter, only one in five patients answered these questionnaires at any time point during their disease trajectory. Missing HRQoL data is more common in clinical studies with patients with glioblastoma.^53^ However, in our study, patients who answered any HRQoL questionnaire might have been fitter than those who did not answer any HRQoL questionnaire when comparing received treatments and performance scores. These differences are the most plausible explanation for the apparently better functioning scores in the subgroup receiving potentially inappropriate care. The reported HRQoL data is hypothesis-generating only and should not be interpreted as a protective effect of receiving potentially inappropriate care. These nuances should be considered when interpreting the HRQoL data reported, especially as the reported HRQoL data is exploratory only. Despite these limitations, our study provides a comprehensive overview of end-of-life care in patients with glioblastoma.

This study gives insight into the prevalence and determinants of potentially inappropriate care at the end of life on a population level. Although the proportion of patients who received potentially inappropriate care at the end of life in this cohort was lower than that reported in other studies, 1 in 5 patients received potentially inappropriate care in the final 3 months of life and ensuring goal-concordant care delivery remains important. Clinicians should discuss the preferences of patients and their relatives regarding care at the end-of-life phase, preferably early in the disease trajectory when patients still have the capacity to engage in these conversations. Structured approaches to these conversations, such as a nurse-led advance care planning program introduced early in the disease trajectory, have been associated with fewer care transitions in the final months of life and greater proxy involvement in end-of-life decision-making in patients with glioblastoma.^19^ In our cohort, a specialist palliative care consultation was recorded for 25 patients, and in more than one third of these patients the consultation took place in the final 30 days of life. This suggests that specialist input is sought often once the end-of-life phase is already apparent. Introducing structured advance care planning conversations at diagnosis and repeating them at first progression, while decision-making capacity is usually still intact, may therefore be more effective than referral triggered by clinical deterioration. In regions where palliative care is conditional on discontinuing anticancer treatment, the use of chemotherapy, which is the largest driver of potentially inappropriate care in our cohort, may conversely be suppressed. This illustrates how differences in how palliative care is organized may also affect both the overall proportion of potentially inappropriate care and which indicators contribute to its receipt. In addition to goal-concordant care delivery in the clinic, future studies should aim to assess the appropriateness of care as accurately as possible, ideally supported by a uniform population-based definition of potentially inappropriate care for patients with a primary malignant brain tumor that accounts for the aggressiveness or burden of interventions. Alternatively, the proportion of patients receiving goal-concordant care could be estimated by comparing preferences documented in medical records with the care actually delivered across care facilities. Delivering goal-concordant care and pragmatic assessment of care at the end of life are important strategies to improve care. Implementing these strategies allows monitoring of goal-concordant care delivery, ultimately reducing the delivery of inappropriate care to patients with glioblastoma and improving wellbeing in these patients and their relatives while simultaneously allowing earlier and more targeted palliative care interventions.

## Supplementary Material

noag198_Supplementary_Data

## Data Availability

The data that support the findings of this study are available from the corresponding author on reasonable request. The data are not publicly available due to privacy restrictions.
