# Supplementary material for: Potentially inappropriate care at the end of life in patients with glioblastoma: A single-center observational cohort study
Source: Neuro Oncol. 2026 Sep 23;28(11):2597–608. doi: 10.1093/neuonc/noag198 (PMC13731850; doi:10.1093/neuonc/noag198)
Supplement: Supplementary file 1 [file noag198_supplementary_data.docx]

**Appendix**

**Appendix Figure 1. Kaplan-Meier curve for survival.** All patients in the cohort are included (n=310).

**Appendix: Table 1. Combinations of indicators.** Table showing the combinations of indicators within individual patients for the final 3 months and the final 30 days of life. N.A. not applicable.

| **Combinations of indicators in patients** | **Number of patients** | |
| --- | --- | --- |
|  | **Final 3 months** | **Final 30 days** |
| No potentially inappropriate care in the final 30 days of life | N.A. | 48 |
| Chemotherapy only | 49 | 8 |
| Chemotherapy and in-hospital death | 2 | 0 |
| Hospital admission for >14 consecutive days | 3 | 2 |
| Hospital admission for >14 consecutive days, ICU admission, chemotherapy, and in-hospital death | 1 | 0 |
| Hospital admission for >14 consecutive days, ICU admission, and in-hospital death | 0 | 1 |
| Hospital admission for >14 consecutive days, and in-hospital death | 0 | 1 |
| ≥2 hospital admissions only | 2 | 0 |
| ≥2 hospital admissions and chemotherapy | 2 | 0 |
| ≥2 hospital admissions, ICU admission, chemotherapy, and in-hospital death | 1 | 0 |
| ≥2 hospital admissions and in-hospital death | 1 | 0 |
| ICU admission, chemotherapy, and in-hospital death | 0 | 1 |
| ICU admission and in-hospital death | 0 | 1 |
| In-hospital death only | 1 | 4 |
| ≥2 ED visits and chemotherapy | 1 | 0 |
| ≥2 ED visits, ≥2 hospital admissions, and chemotherapy | 1 | 0 |
| ≥2 ED visits, ≥2 hospital admissions, hospital admission for >14 consecutive days, and in-hospital death | 1 | 0 |
| ≥2 ED visits, ≥2 hospital admissions, ICU admission, chemotherapy, and in-hospital death | 1 | 0 |
| ≥2 ED visits, ≥2 hospital admissions, and in-hospital death | 1 | 1 |

**Appendix: Table 2. Characteristics of patients with HRQoL data.** Table showing the patient characteristics of those with HRQoL data available. Displayed are n (%) and median (IQR), were appropriate.

|  | **Overall** | **Final 3 months** | | **Final 30 days** | |
| --- | --- | --- | --- | --- | --- |
|  |  | **Potentially appropriate care** | **Potentially inappropriate care** | **Potentially appropriate care** | **Potentially inappropriate care** |
|  | **n=66 (100%)** | **n=54 (82%)** | **n=12 (18%)** | **n=64 (97%)** | **n=2 (3%)** |
| **Male gender [n (%)]** | 42 (64%) | 36 (67%) | 6 (50%) | 42 (66%) | 0 (0%) |
| **Age at diagnosis, in years [median (IQR)]** | 66 (56-72) | 66 (56-71) | 64 (59-73) | 66 (56-72) | 74 (68-80) |
| **Baseline KPS [n (%)]** | | | | | |
| KPS >=70 | 61 (92%) | 50 (93%) | 11 (92%) | 60 (94%) | 1 (50%) |
| KPS <70 | 5 (7.6%) | 4 (7.4%) | 1 (8.3%) | 4 (6.3%) | 1 (50%) |
| **MGMT promoter methylation [n (%)]** | | | | | |
| Methylated | 25 (38%) | 18 (33%) | 7 (58%) | 24 (38%) | 1 (50%) |
| Non-methylated | 41 (62%) | 36 (67%) | 5 (42%) | 40 (63%) | 1 (50%) |
| **Localization [n (%)]** | | | | | |
| Frontal | 20 (30%) | 16 (30%) | 4 (33%) | 20 (31%) | 0 (0%) |
| Midline | 1 (1.5%) | 1 (1.9%) | 0 (0%) | 1 (1.6%) | 0 (0%) |
| Occipital | 1 (1.5%) | 0 (0%) | 1 (8.3%) | 1 (1.6%) | 0 (0%) |
| Parietal | 14 (21%) | 13 (24%) | 1 (8.3%) | 14 (22%) | 0 (0%) |
| Temporal | 30 (45%) | 24 (44%) | 6 (50%) | 28 (44%) | 2 (100%) |
| **Surgery [n (%)]** | | | | | |
| Biopsy only | 10 (15%) | 8 (15%) | 2 (17%) | 10 (16%) | 0 (0%) |
| Resection | 56 (85%) | 46 (85%) | 10 (83%) | 54 (84%) | 2 (100%) |
| **Treatment [n (%)]** | | | | | |
| Chemotherapy | 4 (6.1%) | 0 (0%) | 4 (33%) | 3 (4.7%) | 1 (50%) |
| Chemo- and radiotherapy | 49 (74%) | 43 (80%) | 6 (50%) | 49 (77%) | 0 (0%) |
| Radiotherapy | 13 (20%) | 11 (20%) | 2 (17%) | 12 (19%) | 1 (50%) |
| **Type of chemotherapy (n=53) [n (%)]** | | | | | |
| Lomustine and temozolomide | 17 (32%) | 16 (37%) | 1 (10%) | 17 (33%) | 0 (0%) |
| Temozolomide | 36 (68%) | 27 (63%) | 9 (90%) | 35 (67%) | 1 (100%) |
